# Varicella-zoster virus vasculopathy: a rare complication of Ramsay Hunt Syndrome: a literature review

**DOI:** 10.3389/fneur.2025.1509110

**Published:** 2025-06-19

**Authors:** Di Wu, Mian Song

**Affiliations:** Department of Nursing, Shengjing Hospital of China Medical University, Shenyang, Liaoning, China

**Keywords:** Ramsay Hunt Syndrome, herpes zoster oticus, varicella zoster virus, stroke, VZV vasculopathy

## Abstract

**Background:**

Ramsay Hunt Syndrome TypeII (RHSII), also known as herpes zoster oticus, is an example of reactivation of the varicella-zoster virus (VZV) at the geniculate ganglion. Although rare, reports of VZV vasculopathy secondary to RHSII have been documented. The aim of this review was to analyze clinical and neuroimaging findings, laboratory findings, treatment and outcome of these patients.

**Methods:**

We report a literature review that includes all case reports identified via PubMed on RHSII complicated by VZV Vasculopathy. All epidemiological, clinical, imaging, virologic, treatment and outcome data collected are described.

**Results:**

We analyzed a total of RHSII complicated by VZV vasculopathy 9 cases (median age 54.0 ± 16.5 years, range 24–75), and 22.2% (2/9) were immunocompromised. Apart from RHSII-related symptoms, the most common symptom is cranial nerve injury (77.8%, 7/9). Abnormal brain imaging in 88.9% (8/9), including 8 cases of ischemic stroke and one of which was accompanied by hemorrhagic stroke. Vascular studies revealing abnormalities in 66.7% (6/9) cases. Cerebrospinal fluid (CSF) analysis revealed 5 (55.6%, 5/9) cases were positive for VZV IgG, and 5 (55.6%, 5/9) cases tested positive for VZV DNA. All 9 patients received acyclovir treatment, with 77.8% (7/9) combination with corticosteroids. A favorable outcome was observed in 88.9% (8/9) of the patients. We analyzed a total of 9 cases of RHS II complicated by VZV vasculopathy. The median age was 54.0 ± 16.5 years (range 24–75), with 22.2% (2/9) being immunocompromised. In addition to RHS II-related symptoms, cranial nerve injury was the most common symptom, occurring in 77.8% (7/9) of cases. Abnormal brain imaging was observed in 88.9% (8/9) of patients, including 8 cases of ischemic stroke, one of which was accompanied by hemorrhagic stroke. Vascular studies revealed abnormalities in 66.7% (6/9) of cases. Cerebrospinal fluid (CSF) analysis showed that 5 patients (55.6%) were positive for VZV IgG, and 5 patients (55.6%) tested positive for VZV DNA. All 9 patients received acyclovir treatment, with 77.8% (7/9) also receiving corticosteroids. A favorable outcome was observed in 88.9% (8/9) of the patients.

**Conclusion:**

VZV vasculopathy, as a rare complication of RHSII, can occur in both immunocompetent and immunosuppressed patients. Ophthalmoplegia is its primary clinical manifestation. Detection of VZV IgG antibodies and VZV DNA in the CSF has equal sensitivity. Antiviral combined with steroid therapy represents the optimal treatment approach.

## Introduction

Varicella zoster virus (VZV) is a double-stranded DNA neurotrophic alpha-herpesvirus that replicates almost exclusively in human cells and tissues (1). Primary infection occurs mostly during childhood and presents as varicella (chickenpox). Afterward, the virus may retrogradely travel to the sensory neuron cell bodies within the ganglion, where it establishes a latent infection. Any immune instability or deficiency (such as chemotherapy, tumors, stress, etc.) can trigger the virus to reactivate and replicate. Subsequently, it travels via anterograde axonal transport to the dermatome associated with the affected ganglion, resulting in the development of herpes zoster (2). Ramsay Hunt Syndrome TypeII (RHSII), is an example of reactivation of the VZV at the geniculate ganglion, characterized by ipsilateral facial paralysis, otic pain, erythematous vesicular rash on the ear or in the mouth. Unlike Bell’s palsy, RHSII has a worse prognosis and often results in facial paralysis and postherpetic neuralgia (3). In some cases, VZV can also spread to the arteries of the central nervous system, eventually leading to a uncommon but well-known complication called VZV vasculopathy (4). This was first recognized based on the occurrence of contralateral hemiplegia after trigeminal distribution zoster caused disease to the cerebral arteries (5). Given the significant increase in stroke risk during the first year following herpes zoster infection, researchers have come to realize that VZV vasculopathy may be more common than previously thought (6, 7). Therefore, although VZV vasculopathy is rarely observed in association with RHSII, it is a complication that must not be overlooked, particularly given the treatability of VZV vasculopathy (8). Here, we reviewed the literature and found a total of 9 cases of adult-onset RHSIIaccompanied by VZV vasculopathy, aiming to illustrate the clinical presentations, imaging features, virologic findings, treatment approaches, and outcomes (9–17). To our knowledge, no previous research has addressed this topic, and we believe that this review provides valuable insights for clinicians, aiding in the early recognition and treatment of these complex cases, thereby improving patient outcomes.

## Materials and methods

**Figure 1:**
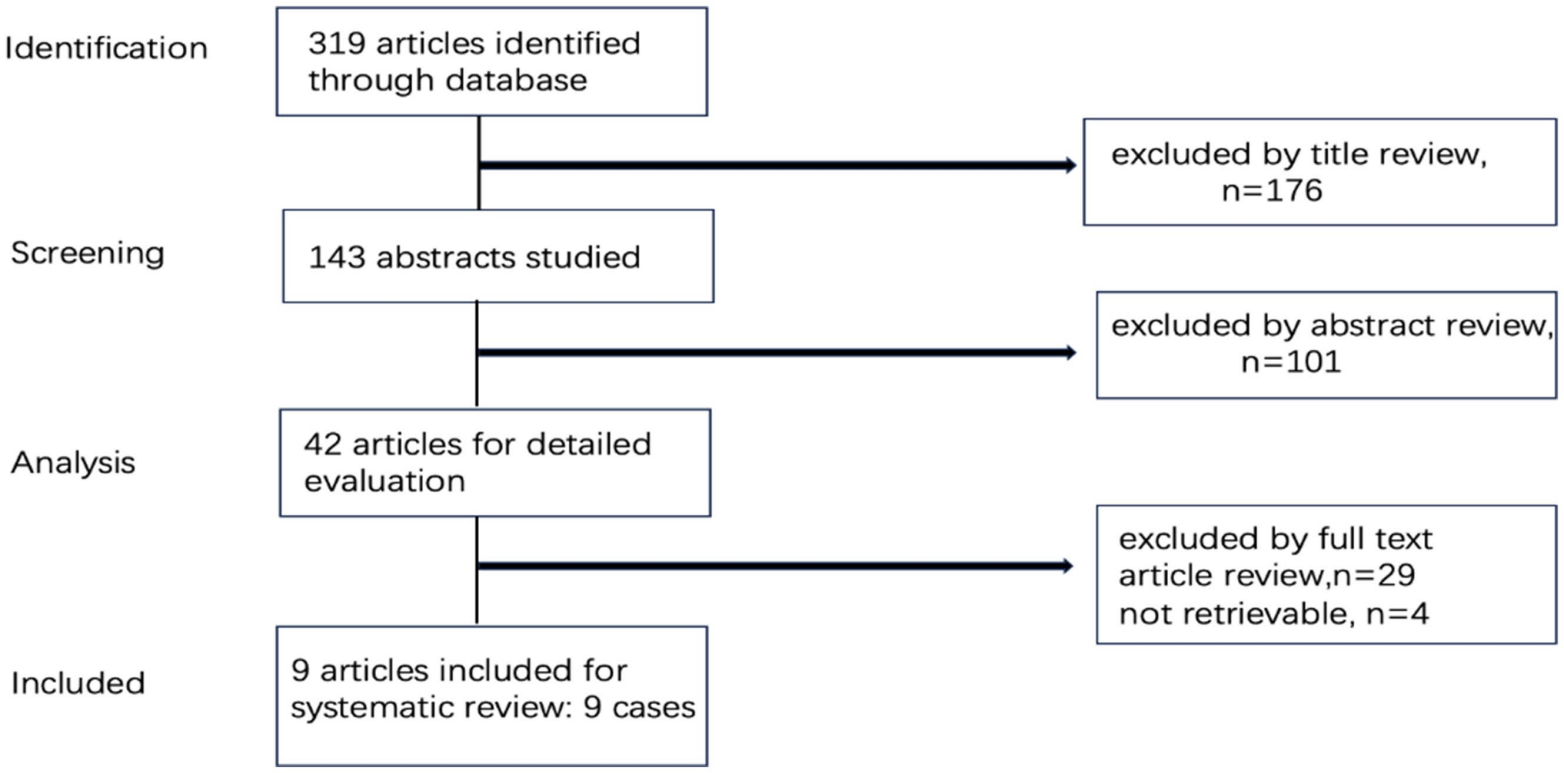
Flow chart of patient selection.

We performed a comprehensive literature review up to 2024, encompassing all case reports retrieved from PubMed using the search terms “VZV vasculopathy,” “contralateral hemiplegia,” “stroke,” “ischemia,” “infarction,” “hemorrhage” and “varicella-zoster virus,” “herpes zoster oticus” or “Ramsay Hunt Syndrome.” Case reports cited in the collected articles were added to the list. Articles written in English were included. We included all epidemiological, clinical, imaging, Cerebrospinal fluid (CSF) test, treatment, and outcome. RHSIIand VZV vasculopathy were confirmed according to diagnostic criteria by consensus article (3, 8). We excluded patients who had incomplete medical histories and imaging data (Figure 1). Given that this was a review of published literature, there was no requirement for an ethics board approval. Based on brain imaging of the cases, arterial disease was classified as large artery, small artery or mixed. The Large arteries disease refers to involvement of the internal carotid artery, the anterior cerebral artery and the middle cerebral artery in the anterior circulation, and the posterior cerebral artery, the vertebral artery and basilar artery in the posterior circulation. In contrast, diseases of the penetrating arteries (e.g., the lenticulostriate arteries) supplying the deep-seated structures were considered small artery diseases (18). We used the Modified Rankin Scale (mRS) as the outcome assessment measure. The definition of poor outcome was mRS greater than or equal to 3 points, while good outcome defined as mRS score of 0–2 points.

## Results

In the 9 articles reviewed, we identified 9 patients with a clinical diagnosis consistent with RHS II complicated with VZV vasculopathy. The epidemiological, and clinical, imaging, virologic, treatment and outcome, are summarized in Table 1. The average age of the 9 patients was 54.0 ± 16.5 years, ranging from 24 to 75 years, with a male-to-female ratio of 4:5. Two patients were immunosuppressed: one due to HIV infection, and the other due to oral corticosteroid therapy for CREST syndrome. Only one patient reported a history of prior herpes zoster infection. In the clinical presentation of RHSII, only 55.6% (5/9) presented with vesicular rash, while auditory changes were the most common accompanying symptom (44.4%,4/9), followed by dizziness, nystagmus (both 22.2%, 2/9). Other central nervous system clinical features include ophthalmoplegia (55.6%, 5/9), abnormal facial sensation (33.3%, 3/9), dysarthria/dysphagia (22.2%, 2/9), ataxia (33.3%, 3/9), hemiparesis (22.2%, 2/9) and signs of encephalitis (33.3%, 3/9).

**Table 1:** The characteristics of 9 varicella-zoster virus vasculopathy after Ramsay Hunt Syndrome cases.

Reference	Gender, age (years)	Comorbidity	History of chicken or vaccination against VZV	Clinical features	Time from symptom onset to MRI	MRI abnormalities	Affected vessel	CSF findings	IV or oral (days)	Outcome (mRS)
Chan et al. (11)	Female, 55	Diabetes, Hypertension, Hyperlipidemia	No	Right ear vesicular rash, right peripheral facial palsy, diplopia, gait and limb ataxia,	6 weeks	Ischemic lesions in left superior occipital	Left posterior cerebral artery	Elevation protein, cells 700 × 10^6^/L (lymphocyte-predominant), VZV PCR (+)	IV acyclovir 21 days, oral prednisone 6 weeks	Facial palsy completely resolved, Significant improvement in cerebellar symptoms (1)
Calabria et al. (10)	Female, 67	Diabetes, Hypertension	No	Left ear vesicular rash, left peripheral facial palsy, left facial pain, gait and limb ataxia	14 days	Ischemic lesions in left pons, left midbrain, right periventricular area	Normal	Elevation protein, cells 40 × 10^6^/L (lymphocyte predominant), VZV PCR (+), VZV IgG (+)	IV acyclovir 10 days	Mild facial weakness (1)
Labbad et al. (13)	Female, 41	No	Chickenpox	Right peripheral facial palsy, right sided deafness, right oculomotor nerve palsy	1 year	Ischemic lesions in right occipital and temporal lobes	Normal	Elevation protein, cells 20 × 10^6^/L (lymphocyte-predominant), VZV IgG (+)	IV acyclovir14 days	Mild facial weakness and deafness, oculomotor nerve palsy (2)
Upreti and Rathod (17)	Female, 45	No	No	Left ear vesicular rash, left sided deafness, left peripheral facial palsy, reduced facial sensation of left, voice change, mild dysphagia, absent eyeball abduction	7 days	Normal	Left internal carotid artery	Biochemistry and cytology was normal, VZV IgG (+)	IV acyclovir 10 days oral prednisolone 20 days antiplatelet	Mild facial weakness, hearing loss persisted (1)
González-Otárula. (12)	Male, 54	No	No	Left peripheral facial palsy, left sided deafness, horizontal nystagmus, dizzy	3 days	Ischemic lesions in left vermis, left pons and medium cerebellar peduncle, right striatum nuclei	Anterior cerebral arteries posterior cerebral artery	Elevation protein, without pleocytosis, VZV PCR (+)	IV acyclovir 14 days methylprednisolone 5 days	Complete recovery (0)
Ortiz et al. (15)	Female, 24	HIV	No	Left ear vesicular rash, left peripheral facial palsy, headache, mild dysarthria, mild dysphagia, left hemiparesis	1 month	Ischemic lesions in right pontine	Vertebral and basilar artery left internal carotid artery left middle cerebral artery left anterior cerebral artery	Elevation protein, cells 9 × 10^6^/L (lymphocyte-predominant), VZV PCR (+)	IV acyclovir 7 days (oral 8 weeks) IV methylprednisolone 7 days (oral 8 week)	Complete recovery (0)
Ohtomo et al. (14)	Male, 75	Prostate cancer (chemotherapy)	No	Left ear vesicular rash, left peripheral facial palsy, fever, headache, nuchal stiffness	2 weeks	Ischemic lesions in in right insula; microbleeding in the left anterior cingulate gyrus and the midbrain interpeduncular fossa.	Normal	Elevation protein, cells 640 × 10^6^/L (neutrophils-predominant) VZV PCR (+), IgM (+)	IV acyclovir 14 days (oral 2 months) oral dexamethasone 2 months	Complete recovery (0)
Russman et al. (16)	Male, 51	CREST syndrome (oral prednisone)	No	Right peripheral facial palsy, right sided deafness, dizzy, confusion, left hemiparesis, ataxia, nystagmus, left oculomotor nerve palsy, seizure	1 month	Ischemic lesions in brainstem, thalamus, caudate nucleus, occipital, parietal, frontal lobes and left temporal lobe	Left middle cerebral artery right posterior cerebral artery	Biochemistry and cytology was normal, VZV IgG (+)	Ivacyclovir 22 days methylprednisolone 31 days	Decreased arousal, spastic quadriparesis, severe dysphagia, tracheostomy dependence (5)
Bilodeau et al. (9)	Male, 74	Hypertension, Hyperlipidemia	No	Right peripheral facial palsy, mild bilateral abducens palsies, loss of consciousness event	3 days	Ischemic lesions in right frontal	Left middle cerebral artery right middle cerebral artery	Elevation protein, cells 47 × 10^6^/L (lymphocyte-predominant), VZV IgG (+)	IV and oral acyclovir 14 days, oral prednisone 14 days	Persistent right facial weakness (1)

The average time from symptom onset to MRI scan is 14 days. 88.9% (8/9) of the cases showed abnormal brain imaging results, including 8 cases of ischemic stroke, one of which also presented with microbleeds unrelated to the ischemic stroke. Among the ischemic stroke cases, 3 (37.5%, 3/8) had isolated lesions, 5 (63.5%, 5/8) had multifocal lesions. All 9 patients underwent vascular studies, revealing vascular abnormalities in 6 cases. The predominant findings were mixed arterial disease (5/9), small artery disease or large artery disease less commonly (2/9). Combined involvement of both anterior and posterior circulation was the most common pattern (66.7%, 6/9), followed by isolated anterior circulation involvement (22.2%, 2/9), with isolated posterior circulation being the least frequent (11.1%, 1/9). CSF analysis revealed pleocytosis in 66.7% (6/9) of patients. Among the cohort, 5 patients tested positive for VZV IgG antibodies, and 5 tested positive for VZV DNA by PCR; both markers were concurrently positive in 2 cases. All patients received acyclovir therapy, either as monotherapy (22.2%, 2/9) or in combination with corticosteroids (77.8%, 7/9). Only one patient received antiplatelet therapy. A favorable clinical outcome was observed in 88.9% (8/9) of patients (Table 2).

**Table 2:** Demographics, clinical features, imaging abnormalities, and outcome.

Demographics (*n* = 9)		
Median age, years	54.0 ± 16.5	(24–75)
Male gender	44.4%	4/9
History of varicella or zoster	11.1%	1/9
Immunosuppression	22.2%	2/9
Diabetes	22.2%	2/9
Ramsay Hunt Syndrome		
Vesicular rash	55.6%	5/9
Deafness	44.4%	4/9
Dizzy	22.2%	2/9
Nystagmus	22.2%	2/9
Other CNS clinical features		
Encephalitis	33.3%	3/9
Hemiplegia	22.2%	2/9
Ataxia	33.3%	3/9
Cranial nerve injury	77.8%	7/9
Abnormal facial sensation (*N* = 3)		
Dysarthria/dysphagia (*N* = 2)		
Extraocular muscle paralysis (*N* = 5)		
Diagnostic testing		
PCR positive for VZV (CSF)	55.6%	5/9
Anti-VZV IgG positive for VZV (CSF)	55.6%	5/9
Both PCR and anti-VZV IgG positive for VZV (CSF)	22.2%	2/9
Anti-VZV IgM positive for VZV (CSF)	11.1%	1/9
CSF findings		
Pleocytosis (> 4 cells/μl)	66.7%	6/9
Elevation protein	77.8%	7/9
Neuroimaging		
Days from symptom onset to MRI (IQR)	14.0	(3–365)
Evidence for stroke	88.9%	8/9
Ischemic stroke (*N* = 8)		
Hemorrhagic stroke (*N* = 1)		
Distribution of lesions (*N* = 8)		
Single	37.5%	3/8
Multiple	62.5%	5/8
Affected areas of circulation		
Anterior	22.2%	2/9
Posterior	11.1%	1/9
Mixed	66.7%	6/9
Evidence for vasculitis by angiography	66.7%	6/9
Small-sized vessels (*N* = 2)		
Large-sized vessels (*N* = 2)		
small and large vessel (*N* = 5)		
Treatment		
Acyclovir treatment alone	22.2%	2/9
Acyclovir + steroid treatment	77.8%	7/9
Anti-platelet medication	11.1%	1/9
Outcome		
Good outcome (mRS 0–2)	88.9%	8/9
Unfavorable outcome (mRS 3–5)	11.1%	1/9

## Discussion

This literature review of nine cases of Ramsay Hunt Syndrome Type II (RHS II) complicated by varicella-zoster virus (VZV) vasculopathy provides critical insights into its clinical presentation, neuroimaging findings, diagnostic approaches, and therapeutic outcomes. The findings demonstrate that VZV vasculopathy can occur in both immunocompetent and immunocompromised patients, with a median age of 54 years. Cranial nerve dysfunction, particularly ophthalmoplegia, emerged as the predominant clinical feature (77.8%), while neuroimaging revealed abnormalities in 88.9% of cases, predominantly ischemic stroke (8/9 cases). Vascular abnormalities were identified in 66.7% of cases, and cerebrospinal fluid (CSF) analysis showed equivalent sensitivity for VZV IgG antibodies and VZV DNA (55.6% each). Treatment with acyclovir, frequently combined with corticosteroids (77.8%), yielded favorable outcomes in 88.9% of patients, underscoring the effectiveness of combined antiviral and corticosteroid therapy in managing this rare yet severe complication of RHS II.

Following primary chickenpox infection, VZV can remain dormant in the host’s ganglia for extended periods. When the immune status changes, VZV can reactivate, leading to central nervous system complications such as RHS II and VZV vasculopathy (2–4). Current research indicates that immunocompromised is a significant risk factor for VZV reactivation, and these patients are more susceptible to developing complications and have a more severe clinical picture (2). Therefore, we have reason to speculate that immunocompromise may be more pronounced when RHS II and VZV vasculopathy coexist. However, similar to previous studies (19), our results show that only 22.2% of the patients were immunosuppressed. This counterintuitive result may be related to genetic susceptibility factors. For instance, Sironi et al. (20) proposed that mutations in toll-like receptor 3 or its pathway might increase the risk of viral dissemination into the central nervous system. Another plausible explanation is the impairment of the immune system due to aging. In our review, the average age was 54.0 ± 16.5 years, consistent with findings from studies on RHS II with concurrent VZV encephalitis (19), which were notably higher than patients with RHS II alone (21). Additionally, Studies have found that diabetic patients are more susceptible to herpes zoster, with a risk 1.6 times higher than that of non-diabetic patients (22, 44). Furthermore, even in diabetic patients who have never had herpes zoster, VZV antigen can still be detected in the adventitia of cerebral arteries, along with vascular lesions (23). This indicates that diabetes is an important risk factor for VZV vasculopathy. In this study, 2 out of 7 immunocompetent patients had diabetes, further supporting the role of diabetes as a significant risk factor for both herpes zoster and VZV vasculopathy.

Although the appearance of vesicular rash helps in the diagnosis of RHS II, it is not a necessary condition. Current research has found that up to 30% of RHS II cases do not exhibit vesicular rash throughout the entire course (3), which is consistent with our findings. RHS II is characterized by herpetic inflammation of the geniculate ganglion of the facial nerve, sometimes accompanied by involvement of the vestibulocochlear nerve, leading to symptoms such as vertigo, nystagmus, tinnitus, hearing loss, hyperacusis, nausea, and vomiting (43). Additionally, the V, VI, IX, X, XI, and XII cranial nerves are occasionally affected, causing ophthalmoplegia, facial sensorimotor changes, bulbar dysfunction, and neck weakness (3, 24, 25). The mechanism by which RHS II leads to multiple intracranial neuropathies and vascular damage is not yet fully understood. Currently, several potential mechanisms have been proposed to explain how the virus spreads to intracranial blood vessels and nerves. Firstly, anatomical proximity may facilitate the spread of the virus between adjacent nerves both inside and outside the cranium, such as the anastomotic connection between the auricular branch of the vagus nerve and the facial nerve in the external auditory canal, as well as the anastomotic connection between the glossopharyngeal nerve and the tympanic membrane. Additionally, the facial nerve passes through the dura mater on its way out from the brainstem nucleus, which could allow for direct viral spread between the nerves and the meninges (24, 26). Secondly, CSF and the external lymphatic fluid of the inner ear are thought to play a role in the transmission of the virus between the facial nerve and cochlear nerve (27). Furthermore, the blood supply between the internal carotid artery, meningeal arteries, and pharyngeal ascending artery may enable the virus to spread hematogenously between multiple cranial nerves (28, 29). Lastly, dysregulated immune modulation proteins and chemokines resulting from abnormal activation of the post-infection defense mechanisms are also thought to be involved in the pathogenesis of VZV-induced neurological diseases (30). In our review, we found that, apart from RHS II-related symptoms, extraocular muscle paralysis was the most prominent symptom. This is inconsistent with previous findings that the glossopharyngeal and vagus nerves are most commonly affected in RHS II patients with intracranial nerves damage (31, 32). This further highlights how the combination of different mechanisms leads to the diversity of clinical symptoms. In addition, we found that 33.3% of patients experienced ataxia. Previous studies have shown that the occurrence of ataxia in RHS II often indicates the presence of acute cerebellitis (33, 34). These findings suggest that when patients with RHS II present with symptoms such as extraocular muscle paralysis or ataxia, careful consideration should be given to identifying potential intracranial complications, particularly VZV vasculopathy. In the past few decades, there has been a deep understanding of the clinical spectrum of VZV vasculopathy, which is prominently characterized by multiple ischemic strokes involving both the anterior and posterior circulations. Vascular examinations revealed that the involvement of both large and small arteries was more common than that of either alone (8). This is consistent with the findings of our review.

Confirmatory laboratory diagnosis of VZV vasculopathy is made by the presence of VZV DNA or anti-VZV antibody in the CSF. However, as time progresses and the disease advances, IgM and VZV DNA levels decrease, making VZV IgG detection the sole diagnostic strategy (8, 35). As suggested in previous reports, our review also emphasizes that the detection of VZV IgG antibodies in CSF has higher sensitivity for diagnosing VZV vasculopathy in patients with RHS II. However, the similar positivity rates of VZV IgG and VZV DNA further emphasize the short interval from RHS II to VZV vasculopathy. Consistent with our review results, routine CSF examinations for VZV vasculopathy are not specific and often indicate mononuclear pleocytosis, increased protein levels, and normal glucose levels (8). In recent years, some researchers have found that CSF levels of IL-8, IL-6, and MMP-2 are significantly elevated in patients with VZV vasculopathy (36). Since IL-8 is a chemotactic factor for neutrophils and IL-6 promotes macrophage differentiation, these findings may help explain the abundance of neutrophils and macrophages observed in patients with VZV vasculopathy.

Antiviral treatment has been widely accepted as the first-line therapeutic approach for VZV infections, although the optimal choice of drug, dose, and duration of treatment are not firmly established (8). Currently, the main focus of discussions regarding the treatment of VZV central nervous system infections centers on whether antiviral therapy should be combined with corticosteroids. Some studies suggest that combination therapy results in better prognosis for patients (3, 8), which is consistent with our findings. However, Wu et al. (37) study suggests that there is no significant difference in mortality or good prognosis between using antiviral drugs alone and combining antiviral drugs with corticosteroids, indicating that the role of glucocorticoids in the treatment of VZV vasculopathy remains unclear. Current research on the treatment of VZV vasculopathy highlights a significant gap in the investigation of antiplatelet therapy. While studies on HIV-and tuberculosis-related vasculitis have demonstrated that aspirin can reduce the risk of stroke (38). In our review, one patient received antiplatelet therapy and showed a very good prognosis. However, this patient also received antiviral drugs and steroids. Therefore, the efficacy of antiplatelet drugs in VZV-related ischemic stroke still requires further clinical validation. Due to the lack of specific therapeutic drugs, vaccination, including the live attenuated zoster vaccine (ZVL) and the inactivated adjuvant recombinant zoster vaccine (RZV), has been proven to be one of the most cost-effective strategies for preventing VZV infection (39, 40).

Previous studies have shown that the prognosis of patients with VZV involving the nervous system is related to multiple factors, including the timing of medication initiation, treatment duration, and whether corticosteroids are used in combination. Among these, earlier antiviral treatment is key, the longer the delay in antiviral treatment, the worse the prognosis (41, 42). In this study, VZV vascular disease occurred after the clinical symptoms of RHS II, leading to relatively early application of antiviral drugs, often in combination with corticosteroid treatment, resulting in a generally good prognosis, which is consistent with previous research findings.

## Conclusion

This review conducted an in-depth exploration of the coexistence of VZV vasculopathy and RHS II, revealing several key points. Firstly, VZV vasculopathy, as a rare complication in RHS II patients, has ophthalmoplegia as a prominent symptom. The detection of IgG and PCR DNA in CSF has proven to be effective diagnostic markers for the coexistence of VZV vasculopathy and RHS II. Second, the results indicate that despite the simultaneous occurrence of these two conditions, the combination of antiviral therapy and steroids has achieved favorable outcomes in nearly all patients. This finding not only validates the effectiveness of current treatment strategies but also provides directions for optimizing future therapeutic approaches. Although this review provides valuable insights, we must acknowledge its limitations. Specifically, the potential publication bias in reports of severe cerebrovascular diseases may affect the generalizability of the conclusions. Therefore, we call for larger-scale, evidence-based medical research in the future to draw more meaningful conclusions. In summary, the findings of this review provide important evidence for a deeper understanding of the coexistence of VZV vasculopathy and RHS II, but further research is needed to enhance the understanding of this field.

## Data Availability

The original contributions presented in the study are included in the article/supplementary material, further inquiries can be directed to the corresponding author.
